# Associations Between Food Insecurity and Diet Quality Among Graduate Students and Postdoctoral Trainees in the Health Sciences at a Private University in Boston: A Cross-Sectional Study

**DOI:** 10.1016/j.cdnut.2024.102157

**Published:** 2024-04-10

**Authors:** Nour M Hammad, Meghan Zimmer, Jarvis T Chen, Deirdre K Tobias, Walter C Willett, Cindy W Leung

**Affiliations:** 1 Department of Nutrition, Harvard T.H. Chan School of Public Health, Boston, MA, United States; 2 Department of Social and Behavioral Sciences, Harvard T.H. Chan School of Public Health, Boston, MA, United States; 3 Division of Preventive Medicine, Department of Medicine, Brigham and Women’s Hospital and Harvard Medical School, Boston, MA, United States; 4 Department of Epidemiology, Harvard T. H. Chan School of Public Health, Boston, MA, United States

**Keywords:** food security, diet quality, Prime Diet Quality Screener Score, graduate students, postdoctoral trainees, private universities

## Abstract

**Background:**

Food insecurity is a pivotal determinant of health outcomes. Little evidence exists on the association between food insecurity and health behaviors and outcomes, including diet quality, among graduate students or postdoctoral trainees.

**Objectives:**

This study aimed to examine the association between food insecurity and diet quality among graduate students and postdoctoral trainees at 3 health-focused graduate schools (public health, medical, and dental medicine) within Harvard University.

**Methods:**

Between April and June 2023, 1287 graduate students and 458 postdoctoral trainees at the health-focused schools within Harvard University completed a web-based survey. The primary exposure was food security status, assessed using the United States Household Food Security Survey Module. The primary outcome was diet quality, measured using the 30-day Prime Diet Quality Score screener (ranges from 0 to 126, with higher scores indicating healthier diets). The associations between food insecurity and diet quality were examined using multivariable regression models, adjusting for sociodemographic covariates.

**Results:**

Among graduate students, compared with those with high food security, diet quality was significantly lower among those experiencing marginal food security [β: −4.7; 95% confidence interval (CI): −6.5, −2.9], low food security (β: −5.4; 95% CI: −7.6, −3.3), and very low food security (β: −4.4; 95% CI: −7.4, −1.4). Poor diet quality included lower intake frequencies of vegetables, fruits, beans/peas/soy products, nuts/seeds, poultry, fish, low-fat dairy, and liquid oils, and higher intake frequencies of refined grains/baked products, sugar-sweetened beverages, and fried foods. Among postdoctoral trainees, compared with those with high food security, diet quality was significantly lower among those experiencing low food security (β: −5.1; 95% CI: −8.8, −1.4), and very low food security (β: −5.2; 95% CI: −10.2, −0.2). Poor diet quality included lower intake frequencies of dark green leafy vegetables, other fruits, and whole grains.

**Conclusions:**

Graduate students and postdoctoral trainees who experienced degrees of food insecurity reported lower diet quality. These observations underscore the need for policies and interventions to simultaneously reduce food insecurity and improve diet quality.

## Introduction

Food insecurity, defined as the lack of access to sufficient food for a healthy and active life, is a pivotal determinant of health outcomes among young adults. Many young adults pursuing postsecondary education may experience food insecurity despite coming from well-educated or affluent socioeconomic backgrounds [1]. A growing body of literature suggests that the prevalence of food insecurity in United States universities is substantially higher than the national average of all age and socioeconomic subgroups [2]. This is concerning given the multitude of adverse consequences of food insecurity, including higher risks of psychological distress, loneliness, anxiety, and depression [[3], [4], [5]]. College food insecurity is also associated with lower academic performance, lower graduation rates, and an increased prevalence of food insecurity later in adulthood [[5], [6], [7]]. Some studies have examined the association between college food insecurity and diet quality [[8], [9], [10]]. Their results have shown that students with food insecurity have lower diet quality, including lower intakes of fruits, vegetables, and whole grains and higher intakes of added sugars and sugar-sweetened beverages. In turn, poor diet quality is a risk factor for other suboptimal health behaviors, such as lower physical activity, more screen time [11,12], as well as lower academic achievement [13,14]. Furthermore, poor diet quality is associated with a higher risk of chronic diseases and poorer psychological well-being among adults [[15], [16], [17]]. Previous studies have shown reductions in all-cause mortality, cardiovascular disease and cancer risk and mortality, and metabolic disease risk among adults with a higher diet quality [12,18].

To date, few studies have explored food insecurity in graduate school settings, at private institutions, or among postdoctoral trainees [19,20]. A notable gap also exists on the associations between food insecurity and health behaviors such as attention to dietary quality in these populations. Exploring these associations is essential because graduate and postdoctoral education represents a unique period for young adults with a myriad of challenges that may exacerbate food insecurity as they navigate research-intensive academic spaces. Such challenges include demanding workloads, financial constraints, geographic demands (e.g., moving for temporary positions), and competitive environments [21]. The lack of literature on the association between food insecurity and diet quality among these populations hinders our ability to address the complex intertwine of challenges they face and to mitigate health disparities and inequities within academic settings. The objective of this study is to help bridge the gap in the literature and explore the associations between food insecurity and diet quality of graduate students and postdoctoral trainees enrolled at 3 health-focused graduate schools within a private academic university in Boston, MA, United States.

## Methods

### Data source and analytic sample

This study used a cross-sectional design. We sent a brief online Qualtrics survey to the university e-mail addresses of all currently enrolled graduate students and active postdoctoral trainees (*n* = 3621) at the T.H. Chan School of Public Health, Medical School, and School of Dental Medicine at Harvard University during the end of the Spring (April–June) 2023 academic term. The purpose of the survey was to assess “experiences and well-being on campus.” Participants received an e-mail with a personalized survey link, and ≤3 reminder e-mails over a 3-wk period. Informed consent was obtained electronically at the beginning of the survey. Respondents received a $10 Amazon.com e-gift card on survey completion. The study was approved by the Harvard T.H. Chan School of Public Health institutional review board.

Overall, we received responses from 1443 graduate students and 525 postdoctoral trainees (55% response rate). After excluding 218 respondents with missing data, low-quality responses (e.g., straight-lined responses for all diet quality screener questions), or unreasonable survey completion duration (e.g., duration as low as 10 s), or students enrolled in fully remote programs, the final analytic sample included 1287 graduate students and 458 postdoctoral trainees aged ≥18 y.

### Measures

The 18-item Household Food Security Survey Module from the United States Department of Agriculture was used to measure household food security status [22]. The indicators’ time frame was altered to assess experiences of graduate students and postdoctoral trainees since the beginning of the Fall 2022 semester (late August 2022) rather than the last 12 mo. Affirmative responses to the 18 items for households with children, or the 10 items for households with only adults, were summed to create a total score. Food security status was then operationalized as 4 levels, according to United States Department of Agriculture procedures [23]: high food security (no affirmative responses), marginal food security (1–2 affirmative responses), low food security (3–7 affirmative responses for households with children; 3–5 affirmative responses for households without children), and very low food security (8–18 affirmative responses for households with children; 6–10 affirmative responses for households without children). The term food insecurity encompasses both low and very low food security status.

Diet quality was measured using the 22-component Prime Diet Quality Score in a 30-d food frequency questionnaire format (PDQS-30D) [24]. The 22 components of this screener include 14 healthy components (dark green leafy vegetables; cruciferous vegetables; deep orange vegetables; other vegetables; citrus fruits; deep orange fruits; other fruits; beans, peas, and soy products; nuts and seeds; poultry; fish; low-fat dairy; whole grains; and liquid oils), 7 unhealthy components (white roots and tubers; red meat; processed meat; refined grains and baked products; sugar-sweetened beverages; sweets and ice cream; and fried foods away from home), and 1 neutral component (eggs). The following categories are possible responses that correspond to the frequency of intake of each of these food groups: once a month or less, 2–3 times per month, 1–2 times per week, 3–4 times per week, 5–6 times per week, 1 time per day, or 2 or more times per day. Responses to the healthy components are coded from 0 to 6, unhealthy components are reverse coded, and the neutral component is not coded. A total PDQS-30D diet quality score of range 0–126 is then created by summing the individual component scores, with higher scores reflecting better diet quality.

### Statistical analysis

Descriptive statistics including means and standard deviations were examined for the unadjusted PDQS-30D scores by covariates representing sociodemographic characteristics and food security status. The normality of these scores was assessed visually through histograms and quantitatively through Shapiro–Wilk tests. Next, we conducted 3 sets of linear regression models to examine the associations of food security status with PDQS-30D scores, and both models were analyzed separately for graduate students and postdoctoral trainees. Model 1 was unadjusted. A minimally adjusted model, model 2, was adjusted for age, gender, and race/ethnicity. A fully adjusted model, model 3, was adjusted for age, gender, race/ethnicity, first-generation status, marital status, presence of children, employment status (graduate students model only), receipt of financial aid (graduate students model only), receipt of Supplemental Nutrition Assistance Program (SNAP), housing instability, and car ownership, that is, significant covariates obtained from the descriptive statistics. Trend tests across categories of food security status were calculated using the analysis of variance function from the “Car” package in R. Next, adjusted means of PDQS-30D components by food insecurity status were computed from model 2 (to avoid overfitting) for both graduate students and postdoctoral trainees and tested for significant differences across food insecurity status using Wald tests. Statistical analysis was performed in R version 4.2.2; all tests were 2-sided and significance was considered at *P* < 0.05.

## Results

Mean PDQS-30D scores for graduate students and postdoctoral trainees by sociodemographic characteristics and food security status are summarized in Table 1. The mean (SD) PDQS-30D score was 63.6 (12.1) points for graduate students and 65.2 (10.7) points for postdoctoral trainees, of 126 possible points. Overall, 16.2% of the graduate students experienced marginal food security and another 17.5% reported low or very low food security. Similarly, 15.5% of the postdoctoral trainee sample reported marginal food security and another 12.7% reported low or very low food security (Table 1).

**TABLE 1:** Mean unadjusted PDQS-30D scores by sociodemographic characteristics and food security status of graduate students and postdoctoral trainees at Harvard University.

	Total (*n* = 1745)		Graduate students (*n* = 1287)		Postdoctoral trainees (*n* = 458)	
	*n* (%)	Mean (SD)	*n* (%)	Mean (SD)	*n* (%)	Mean (SD)
Age (y)						
30 and younger	1038 (59.5)	62.9 (11.5)	921 (71.6)	62.9 (11.6)	117 (25.5)	62.9 (10.6)
31–40	624 (35.8)	65.7 (12.0)^1^	300 (23.3)	65.4 (13.2)^1^	324 (70.7)	65.9 (10.7)^1^
41–50	62 (3.6)	65.2 (11.8)	48 (3.7)	64.5 (12.6)	14 (3.1)	67.5 (8.5)
51 and older	21 (1.2)	67.9 (12.7)	18 (1.4)	67.4 (12.3)	3 (0.7)	70.3 (17.9)
Gender						
Male	637 (36.5)	62.8 (11.6)	427 (33.2)	62.5 (12.4)	210 (45.9)	63.4 (9.6)
Female	1073 (61.5)	64.8 (11.9)^1^	830 (64.5)	64.1 (11.9)^1^	243 (53.1)	66.9 (11.4)^1^
Other^2^	35 (2.0)	65.6 (10.5)	30 (2.3)	66.5 (10.7)	5 (1.09)	60.4 (8.5)
Race/ethnicity						
White/Caucasian	625 (35.8)	66.5 (11.3)	441 (34.3)	66.3 (11.6)	184 (40.2)	66.8 (10.6)
Asian	694 (39.8)	62.3 (11.0)^1^	507 (39.4)	62.1 (11.2)^1^	187 (40.8)	63.0 (10.4)^1^
Black/African American	103 (5.9)	61.3 (14.6)^1^	85 (6.6)	60.5 (14.9)^1^	18 (3.9)	65.1 (12.7)
Hispanic	118 (6.8)	64.3 (12.1)	93 (7.2)	63.2 (12.5)^1^	25 (5.5)	68.5 (9.7)
Multiracial/multiethnic	107 (6.1)	65.3 (12.0)	89 (6.9)	64.3 (12.1)	18 (3.9)	70.1 (10.6)
Other^3^	98 (5.6)	62.1 (12.9)^1^	72 (5.6)	61.2 (13.6)^1^	26 (5.7)	64.3 (10.5)
Student/trainee status^4^						
Domestic	1039 (59.5)	64.3 (11.8)	858 (66.7)	63.9 (12.0)	181 (39.5)	65.8 (10.8)
International	662 (37.9)	63.5 (11.7)	420 (32.6)	62.9 (12.2)	242 (52.8)	64.7 (10.5)
Other^5^	43 (2.5)	66.6 (11.7)	9 (0.7)	69.3 (12.9)	34 (7.4)	65.9 (11.5)
Degree type						
Master’s degree	NA	NA	582 (45.2)	63.7 (12.0)	NA	NA
PhD, DMD, or other graduate professional degree	NA	NA	687 (53.4)	63.6 (12.2)	NA	NA
Other^6^	NA	NA	18 (1.4)	63.6 (12.3)	NA	NA
Employed^4^						
Yes	NA	NA	817 (63.5)	63.8 (12.0)	NA	NA
No	NA	NA	468 (36.4)	63.2 (12.2)	NA	NA
Marital status						
Married	507 (29.1)	66.0 (11.3)^1^	285 (22.1)	66.4 (11.8)^1^	222 (48.5)	65.6 (10.7)
Not married	1238 (70.9)	63.2 (11.8)	1002 (77.9)	62.8 (12.1)	236 (51.5)	64.9 (10.8)
Children						
Yes	255 (14.6)	66.9 (10.8)	160 (12.4)	66.5 (11.1)	95 (20.7)	67.5 (10.3)
No	1490 (85.4)	63.6 (11.8)	1127 (87.6)	63.2 (12.2)^1^	363 (79.3)	64.6 (10.8)^1^
First generation						
Yes	370 (21.2)	64.3 (11.5)	232 (18.0)	63.7 (12.2)	138 (30.1)	65.3 (10.0)
No	1375 (78.8)	64.0 (11.8)	1055 (82.0)	63.6 (12.1)	320 (69.9)	65.2 (11.0)
Received financial aid^4^						
Yes	NA	NA	416 (32.3)	63.7 (12.9)	NA	NA
No	NA	NA	870 (67.6)	63.6 (11.7)	NA	NA
Received SNAP						
Yes	80 (4.6)	61.5 (13.6)^1^	72 (5.6)	61.2 (14.2)	8 (1.8)	64.6 (4.1)
No	1665 (95.4)	64.2 (11.6)	1215 (94.4)	63.8 (11.9)	450 (98.3)	65.2 (10.8)
Housing stability						
Yes	1503 (86.1)	64.6 (11.6)	1101 (85.5)	64.3 (11.8)	402 (87.8)	65.4 (11.0)
No	242 (13.9)	60.8 (12.3)^1^	186 (14.5)	59.8 (13.2)^1^	56 (12.2)	64.3 (8.06)
Car ownership						
Yes	465 (26.6)	65.2 (11.4)	319 (24.8)	65.2 (11.9)	146 (31.9)	65.4 (10.3)
No	1280 (73.4)	63.6 (11.8)^1^	968 (75.2)	63.1 (12.1)^1^	312 (68.1)	65.1 (10.9)
Food security status						
High	1183 (67.8)	65.8 (11.6)	854 (66.4)	65.5 (11.9)	329 (71.8)	66.3 (10.8)
Marginal	280 (16.0)	61.4 (10.8)^1^	209 (16.2)	60.6 (10.8)^1^	71 (15.5)	63.7 (10.5)
Low	177 (10.1)	59.5 (10.9)^1^	141 (11.0)	59.2 (11.2)^1^	36 (7.9)	60.7 (9.6)^1^
Very low	105 (6.0)	59.7 (12.7)^1^	83 (6.5)	59.2 (13.7)^1^	22 (4.8)	61.7 (8.4)

Abbreviations: SNAP, Supplemental Nutrition Assistance Program; NA, Not Applicable.

1 *P* < 0.05, indicating statistical significance; *P* values obtained from separate simple linear regressions with PDQS-30D score as the outcome and each categorical variable as the predictor.

2 Includes transgender male, transgender female, neither exclusively male nor female (gender-queer/gender nonconforming), agender, 2-spirit. These categories were collapsed because of the sparsity of data in them to protect the identity of those respondents.

3 Includes Black/African American, Hispanic, Latin, Middle Eastern or North African, Hawaiian or Pacific Islander, Native American or Alaskan Native, and multiracial/multiethnic. These categories were collapsed because of the sparsity of data in them to protect the identity of those respondents.

4 Includes missing data of <0.5%.

5 Includes Green card holders, J1 visa holders, dual citizenships, international but tax residents.

6 Includes residency programs, dental residents, nondegree students/trainees, multiple degrees.

Among graduate students and postdoctoral trainees, significantly higher PDQS-30D scores were observed among those who were aged 31–40 y (compared with 30 y or younger), identified as female (compared with male), identified as non-Hispanic White/Caucasian (compared with graduate students with other racial and ethnic groups; compared with postdoctoral trainees identifying as Asian), and had children (compared with not having children) (all *P* < 0.05). In addition, among graduate students only, significantly higher PDQS-30D scores were observed among those who were married (compared with unmarried), had housing stability (compared with having housing instability), and owned a car (compared with not owning a car) (all *P* < 0.05).

In descriptive analyses, graduate students with marginal food security (score of 60.6), low food security (score of 59.2), and very low food security (score of 59.2) had lower PDQS-30D scores compared with graduate students with high food security (score of 65.5; all *P* < 0.05). Similarly, postdoctoral trainees with marginal food security (score of 63.7; *P* = 0.058), low food security (score of 60.7; *P* < 0.05), and very low food security (score of 61.7; *P* = 0.051) had lower PDQS-30D scores compared with postdoctoral trainees with high food security (score of 66.3).

Unadjusted and multivariable-adjusted associations between food security status and diet quality are summarized in Table 2. Among graduate students, in the unadjusted model 1, those experiencing marginal, low, and very low food security had significantly lower diet quality compared with students experiencing high food security. Similarly, compared with graduate students experiencing high food security, marginal food security [β: −4.7; 95% confidence interval (CI): −6.5, −3.0], low food security (β: −5.8; 95% CI: −7.9, −3.7), and very low food security (β: −5.6; 95% CI: −8.3, −2.9) were significantly associated with lower diet quality, when adjusting for age, gender, and race/ethnicity (model 2). These associations were similar after adjusting for several study covariates (model 3). Among postdoctoral trainees, in unadjusted model 1, those experiencing low food security (β: −5.6; 95% CI: −9.2, −1.9) had significantly lower diet quality than students experiencing high food security. When adjusting for age, gender, and race/ethnicity, and compared with postdoctoral trainees experiencing high food security, low food security (β: −4.3; 95% CI: −7.9, −0.7) continued to be significantly associated with lower diet quality (model 2). This association persisted after adjusting for several study covariates: low food security was associated with a mean 5.1-point lower PDQS-30D score (95% CI: −8.8, −1.4) compared with high food security. Very low food security was also associated with a mean 5.2-point lower PDQS-30D score (95% CI: −10.2, −0.2) than high food security, after adjusting for several covariates (model 3). In both groups, there were significant linear trends for greater food insecurity and lower PDQS-30D scores (*P*-trend < 0.001 for graduate students, *P*-trend < 0.05 for postdoctoral trainees).

**TABLE 2:** Food security status association with diet quality among graduate students and postdoctoral trainees at Harvard University.

	Model 1^1^		Model 2^2^		Model 3^3^	
	β	95% CI	β	95% CI	β	95% CI
Graduate students						
High food security	Reference		Reference		Reference	
Marginal food security	−4.9	−6.7, −3.2	−4.7	−6.5, −3.0	−4.7	−6.5, −2.9
Low food security	−6.4	−8.5, −4.3	−5.8	−7.9, −3.7	−5.4	−7.6, −3.3
Very low food security	−6.4	−9.0, −3.7	−5.6	−8.3, −2.9	−4.4	−7.4, −1.4
*P*-trend^4^	<0.001		<0.001		<0.001	
Adjusted *R*^2^	0.048		0.078		0.085	
Postdoctoral trainees						
High food security	Reference		Reference		Reference	
Marginal food security	−2.6	−5.4, 0.09	−2.0	−4.7, 0.7	−2.2	−5.0, 0.6
Low food security	−5.6	−9.2, −1.9	−4.3	−7.9, −0.7	−5.1	−8.8, −1.4
Very low food security	−4.6	−9.1, 0.01	−4.1	−8.6, 0.5	−5.2	−10.2, −0.2
*P*-trend^4^	0.003		0.028		0.014	
Adjusted *R*^2^	0.023		0.079		0.080	

1 Model 1 unadjusted.

2 Model 2 adjusted for age, gender, and race/ethnicity.

3 Model 3 adjusted for age, gender, race/ethnicity, first-generation status, marital status, presence of children, employment status, receipt of financial aid, receipt of SNAP, housing (in)stability, and car ownership for the graduate students model. In the postdoctoral trainees model, it is adjusted for age, gender, race/ethnicity, first-generation status, marital status, presence of children, receipt of SNAP, housing (in)stability, and car ownership.

4 *P* for linear trend obtained using the analysis of variance function to test for the overall significance of the food security status categories, including testing for linear trend. The type III argument was specified, which is suitable for categorical predictors.

Mean adjusted scores of PDQS-30D individual components by food security status among graduate students and postdoctoral trainees are presented in Table 3. Among graduate students, greater severity of food insecurity was associated with less frequent intakes of key measures of diet quality, including dark green leafy vegetables, cruciferous vegetables, deep orange vegetables, other vegetables, citrus fruits, deep orange fruits, other fruits, beans, peas, and soy products, nuts and seeds, poultry, fish, low-fat dairy, and liquid oils (all *P* < 0.05). Graduate students with marginal, low, and very low food security were found to consume more refined grains and baked products, sugar-sweetened beverages, and fried foods away from home than graduate students with high food security (*P* < 0.05). Among postdoctoral trainees, significant trends were also observed with greater severity of food insecurity being associated with less frequent intakes of dark green leafy vegetables, other fruits, and whole grains (all *P* < 0.05).

**TABLE 3:** Mean adjusted scores of PDQS-30D individual components by food security status among graduate students and postdoctoral trainees at Harvard University.

	Food security status among graduate students (*n* = 1287)				Food security status among postdoctoral trainees (*n* = 458)			
	High (*n* = 854)	Marginal (*n* = 209)	Low and very low (*n* = 224)	*P*^1^	High (*n* = 329)	Marginal (*n* = 71)	Low and very low (*n* = 58)	*P*
Dark green leafy vegetables	3.2 (0.1)	2.7 (0.1)	2.4 (0.2)	<0.001	3.1 (0.1)	2.8 (0.2)	2.5 (0.2)	0.004
Cruciferous vegetables	2.7 (0.1)	2.4 (0.1)	2.1 (0.1)	<0.001	2.3 (0.1)	2.2 (0.2)	1.9 (0.2)	0.162
Deep orange vegetables	2.2 (0.1)	1.8 (0.1)	1.9 (0.1)	<0.001	2.1 (0.1)	1.6 (0.2)	1.9 (0.2)	0.051
Other vegetables	3.6 (0.1)	3.2 (0.1)	2.9 (0.1)	<0.001	3.7 (0.1)	3.6 (0.2)	3.4 (0.2)	0.273
Citrus fruits	2.3 (0.1)	2.0 (0.2)	1.9 (0.2)	<0.001	2.3 (0.2)	2.1 (0.2)	1.9 (0.2)	0.061
Deep orange fruits	1.0 (0.1)	0.9 (0.1)	0.9 (0.1)	0.041	1.0 (0.1)	0.7 (0.2)	0.8 (0.2)	0.246
Other fruits	3.5 (0.1)	3.0 (0.2)	3.0 (0.2)	<0.001	3.4 (0.2)	3.1 (0.2)	2.7 (0.2)	0.019
Beans, peas, and soy products	2.7 (0.1)	2.3 (0.2)	2.3 (0.2)	<0.001	2.5 (0.1)	2.4 (0.2)	2.6 (0.2)	0.847
Nuts and seeds	3.3 (0.1)	2.9 (0.2)	2.8 (0.2)	<0.001	2.9 (0.2)	2.7 (0.2)	2.4 (0.2)	0.075
Poultry	2.5 (0.1)	2.5 (0.2)	2.3 (0.2)	0.008	2.3 (0.1)	2.5 (0.2)	2.2 (0.2)	0.571
Fish	1.5 (0.1)	1.2 (0.1)	1.2 (0.1)	<0.001	1.5 (0.1)	1.3 (0.2)	1.1 (0.2)	0.057
Low-fat dairy	3.1 (0.2)	3.1 (0.2)	2.8 (0.2)	0.006	2.8 (0.2)	2.6 (0.3)	2.3 (0.3)	0.088
Whole grains	3.4 (0.1)	3.3 (0.2)	3.2 (0.2)	0.287	3.5 (0.2)	3.5 (0.2)	3.1 (0.2)	0.004
Liquid oils	3.3 (0.1)	3.0 (0.2)	2.9 (0.2)	0.004	3.8 (0.2)	3.4 (0.2)	3.8 (0.2)	0.064
White roots and tubers	3.9 (0.1)	3.9 (0.1)	3.8 (0.1)	0.254	3.8 (0.1)	3.8 (0.2)	3.9 (0.2)	0.571
Red meat	4.4 (0.1)	4.4 (0.1)	4.6 (0.1)	0.153	4.4 (0.1)	4.7 (0.2)	4.7 (0.2)	0.212
Processed meat	4.9 (0.1)	4.8 (0.1)	4.7 (0.1)	0.057	4.7 (0.1)	4.7 (0.2)	4.7 (0.2)	0.999
Refined grains and baked products	3.2 (0.1)	3.1 (0.2)	3.3 (0.2)	0.011	3.0 (0.1)	3.4 (0.2)	2.9 (0.2)	0.21
Sugar-sweetened beverages	5.3 (0.1)	4.9 (0.1)	5.1 (0.1)	<0.001	5.2 (0.1)	5.3 (0.2)	5.0 (0.2)	0.352
Sweets and ice cream	3.4 (0.1)	3.5 (0.2)	3.6 (0.2)	0.110	3.6 (0.1)	3.5 (0.2)	3.6 (0.2)	0.825
Fried foods away from home	4.9 (0.1)	4.7 (0.1)	4.7 (0.1)	0.010	4.9 (0.1)	4.9 (0.1)	4.9 (0.1)	0.98

All models adjusted for age, gender, and race/ethnicity.

1 *P* obtained from Wald test.

## Discussion

The associations between food insecurity and diet quality among graduate students and postdoctoral trainees at a private academic university in Boston were examined. The findings identified a high prevalence (16%) of food insecurity and overall low diet quality scores (mean of 64.1 points of total 126 possible points) among both graduate students and postdoctoral trainees enrolled at health-focused graduate schools at Harvard University. The mean diet quality scores (as indicated by PDQS-30D) were low among the respondents. However, they are similar to the diet quality scores reported in other graduate student populations, as assessed using other diet quality indexes. For example, the mean diet quality scores in a university dental student population revealed an overall score of 47.3 (of 100 possible points) [25]. Furthermore, it is notable that the mean PDQS-30D scores presented in this study are slightly higher than estimates using PDQS-30D in national samples of low-income United States adults (50.2–51.9 of 126 possible points) [26,27].

Food insecurity was consistently associated with poorer diet quality in both graduate students and postdoctoral trainees. These findings were surprising considering that these populations are highly educated and gaining additional training in the health sciences field, underscoring the notion that food insecurity can be inversely associated with diet quality in all individuals irrespective of their educational background. Most studies assessing food insecurity and diet quality focus on the undergraduate student population. However, previous studies have shown differing characteristics between undergraduate and graduate students in relation to food insecurity, which limits the generalizability of those findings to the exclusively graduate student populations [28,29]. This study fills in the gap by looking distinctly at a graduate student population, with the additional inclusion of postdoctoral trainees, who are also notably underrepresented in this literature.

Graduate students experiencing marginal food security and food insecurity were found to have a lower diet quality than their food-secure counterparts. Low diet quality was characterized by lower intake frequencies of dark green leafy vegetables, cruciferous vegetables, deep orange vegetables, other vegetables, citrus fruits, other fruits, beans, peas, and soy products, nuts and seeds, poultry, fish, low-fat dairy, and liquid oils. Graduate students experiencing marginal, low, and very low food security also had higher intake frequencies of refined grains and baked products, sugar-sweetened beverages, and fried foods away from home compared with their counterparts. These results align with the findings of the systematic review by Shi et al. [10], which found a lower consumption of fruits and vegetables and a higher consumption of sugar-sweetened beverages among food-insecure university students [10]. Similarly, Marshall et al. [18] also found a strong inverse association between food insecurity and diet quality, mainly fruit and vegetable intake along with added sugars, among dental students [18]. The study results are further supported by Sackey et al. [19], who found that having an excellent, very good, or good self-reported diet quality was associated with lower odds of being food-insecure among health sciences graduate students [19]. Collectively, findings from this study and the literature suggest that food insecurity is linked to lower diet quality among graduate students through specific dietary components, especially low fruit and vegetable intake.

There have been virtually no studies of food insecurity among postdoctoral trainees in the growing food insecurity literature of collegiate populations. Given that postdoctoral trainees have already achieved their terminal degree and are at a career inflection point, they have often been overlooked in the college food insecurity literature. However, it is imperative to undertake such studies as postdoctoral trainees face a myriad of hardships in this unique career stage, such as inadequate living stipends to meet the cost of living in their geographic area, financial strain, the lack of a social support network, and the overall competitive research and funding environment [30,31]. The findings of this study show a concerning prevalence of food insecurity among postdoctoral trainees (12.7%). In addition, 15.5% reported marginal food security. Food-insecure and marginally food-secure postdoctoral trainees had lower intake frequencies of dark green leafy vegetables, other fruits, and whole grains than their counterparts. These findings are significant considering the associations of food insecurity and poor nutritional outcomes among adults as described in previous studies [32,33]. Given the observed low intake of fruits and vegetables among both graduate students and postdoctoral trainees, interventions aimed at addressing the particular intake of fruits and vegetables could be useful for both populations. In an effort to understand the experiences of food insecurity among university students, qualitative studies have shown that students viewed food as a last financial priority over other expenses, such as rent, housing expenses, and care for dependents or pets [34,35]. Given the uniqueness of the postdoctoral period and the additional expenses that the aforementioned hardships could incur, future studies are needed to examine the contributors to and consequences of food insecurity among postdoctoral trainees in diverse academic and research settings. Policies are also warranted to raise the salaries of postdoctoral trainees to account for increases in cost of living; these have been approved by the Harvard Chan School faculty and are set to take effect this summer.

Given the robust inverse relationships between food insecurity and diet quality observed in this study, both national food assistance programs and on-campus food pantries could be leveraged to help better alleviate food insecurity and improve diet quality. For example, SNAP is the largest federal nutrition assistance program; however, several barriers have been noted for college students, broadly speaking, in participating in SNAP. One of these is its rigid eligibility criteria, whereby students are required to work for ≥20 h per week to be considered eligible, which may not be legal or feasible with a full course load. For example, graduate students and postdoctoral trainees receiving stipends from the National Institutes of Health are only allowed to work for an additional 25% of their time (∼10 h per week) [36]. Although these policies are in place to protect students and postdoctoral trainees’ ability to progress in their degrees and training, it temporarily restricts their income-earning potential. With <20 h of work per week, graduate students and postdoctoral trainees may be ineligible for SNAP. Other barriers include the confusion about SNAP, its limited outreach to eligible students, and the lack of resources to assist students with their applications. In addition to these structural barriers, students could also refrain from using SNAP because of the social stigma attached to the program [37,38], further exemplifying the need for other wrap-around services that target food security support. Furthermore, on-campus food pantries could also be used to address the short-term needs of graduate students and postdoctoral trainees experiencing food insecurity. Nevertheless, multiple barriers exist for food pantry use as reported in previous literature. Barriers include the lack of awareness of the existing pantry services, insufficient information on policies pertaining to pantry use, inconvenient hours of operation, self-identity (e.g., unavailability of culturally appropriate foods), and social stigma [39,40]. Identifying strategies to address barriers to using national food assistance programs benefits, such as SNAP, and on-campus food pantries is essential. Policies are also needed to structurally address those barriers such as increasing the stipends that graduate students and postdoctoral trainees receive. In fact, collective bargaining through the Harvard Graduate Student Workers Union led to a policy to increase Harvard Kenneth C. Griffin Graduate School of Arts and Sciences PhD stipends, effective at the start of the 2024–2025 academic year [41].

Several resources for food exist for graduate students and postdoctoral trainees to access at the 3 Harvard schools explored in this study [42]. These include discounted meals for both students and trainees available for purchase at the school cafeterias (20% discount) as well as occasional free meals offered at meetings, seminars, and various campus gatherings. In addition, Daily Dollar Deals are available for purchase at Sebastian's Café (Harvard Chan School’s main cafeteria) during breakfast and lunch. There are also multiple, albeit relatively expensive, restaurants and grocery stores that are located within walking distance from the schools. The GRADPLUS declining balance meal plan is also offered for Harvard graduate or professional school students and can be used at Sebastian’s Café [43]. A formal assessment is needed to understand the impact of these resources on food insecurity and to evaluate the quality of foods offered through these resources.

The strengths of this study include its novelty, large sample size, and the use of validated assessment tools for measuring food insecurity and diet quality. The main limitation is the cross-sectional design that limits causal interpretations and directionality of the findings. In addition, the self-reported nature of the data, particularly pertaining to the dietary questions, may lead to social desirability bias. Furthermore, the survey was administered toward the end of the Spring semester when respondents were also burdened with final examinations and other academic or personal responsibilities. Repeating the assessment throughout the academic year could determine whether there are seasonal trends in these measures that we were unable to capture. Finally, because the participants of this study are graduate students and postdoctoral trainees at a private academic institution, the findings might not be generalizable to undergraduate students or students enrolled at public institutions, those in rural or other geographic regions in the United States or in urban areas with lower costs of living. Other studies assessing food insecurity among graduate students at different private universities have shown a similar demographic composition to our study in terms of age, gender, and race/ethnicity [19,44].

In conclusion, food insecurity was associated with lower diet quality among graduate students and postdoctoral trainees enrolled at a private university in Boston, MA. Despite this population’s high educational attainment, the findings demonstrated that when subject to the unique pressures of graduate school and the postdoctoral trainee period with respect to workload, financial constraints, and other hardships, graduate students and postdoctoral trainees alike can struggle to consume adequate quantity and quality of food. Given the adverse and long-term implications of both food insecurity and inadequate diet quality, sustainable interventions are needed to reduce food insecurity and improve diet quality, and their dual burden, in these distinct academic populations. Comprehensive policies aimed at supporting the nutritional health of graduate students and postdoctoral trainees in higher education (e.g, stipend increases, on-campus pantries, emergency resources, and wrap-around support) are warranted.

## Author contributions

The authors’ responsibilities were as follows – NMH, CWL: designed and conducted research; NMH, JTC: analyzed data; NMH: wrote the paper; MZ, DKT, WCW, CWL: made substantial revisions to the content; NMH: had primary responsibility for final content; and all authors: have read and approved the final manuscript.

## Conflict of interest

The authors report no competing interests.

## Funding

This project was funded by the Harvard T.H. Chan School of Public Health Dean’s Office. The funder had no role in study design; collection, analysis, and interpretation of data; and writing of the report and its publication. MZ is supported by T32-DK-007703. The views expressed in this publication are those of the authors and should not be construed to represent any official National Institutes of Health or United States Government determination or policy.

## Data availability

Data described in the manuscript and analytic code will not be made available because of the sensitivity of the data.
